# Transcranial Direct Current Stimulation Enhances Episodic Memory in Healthy Older Adults by Modulating Retrieval-Specific Activation

**DOI:** 10.1155/2020/8883046

**Published:** 2020-12-05

**Authors:** Lijuan Huo, Zhiwei Zheng, Jia Huang, Rui Li, Jin Li, Juan Li

**Affiliations:** ^1^Center on Aging Psychology, CAS Key Laboratory of Mental Health, Institute of Psychology, Chinese Academy of Sciences, Beijing, China; ^2^Department of Psychiatry, Affiliated Brain Hospital of Guangzhou Medical University (Guangzhou Huiai Hospital), Guangzhou, China; ^3^Department of Psychology, University of Chinese Academy of Sciences, Beijing, China; ^4^Inner Mongolia Mental Health Center, Inner Mongolia, China

## Abstract

Memory decline has become an issue of major importance in the aging society. Anodal transcranial direct current stimulation (atDCS) is a viable tool to counteract age-associated episodic memory deterioration. However, the underlying neural mechanisms are unclear. In this single-blind, sham-controlled study, we combined atDCS and functional magnetic resonance imaging to assess the behavioral and neural consequences of multiple-session atDCS in older adults. Forty-nine healthy older adults received either 10 sessions of anodal or sham stimulation over the left dorsolateral prefrontal cortex. Before and after stimulation, participants performed a source memory task in the MRI scanner. Compared to sham stimulation, atDCS significantly improved item memory performance. Additionally, atDCS significantly increased regional brain activity around the stimulation area in the prefrontal cortex and extended to the bilateral anterior cingulate cortex. Neural changes in the prefrontal cortex correlated with memory gains. Our findings therefore indicate that multiple-session offline atDCS may improve memory in older adults by inducing neural alterations.

## 1. Introduction

Transcranial direct current stimulation (tDCS) is a noninvasive technique for brain stimulation. Over the past decade, it has drawn substantial attention in cognitive neuroscience for its potential to improve cognitive function. By delivering a weak direct electrical current (0.5–2 mA) through the scalp, tDCS can directly modulate cortical excitability. Evidence from animal studies and studies on the human motor cortex shows that anodal tDCS (atDCS) may modulate synaptic activity via neurotransmitters and induce synaptic plasticity [1–4]. Long-term potentiation (LTP) is a key process involved in learning and memory. Prolonged atDCS application, especially periodic and repeated stimulation with multiple sessions, is associated with longer-lasting effects resembling LTP mechanisms [5, 6].

Episodic memory refers to the specific memory of past events or experiences [7]. Vivid memory for an episode generally involves remembering individual items (i.e., item memory) and the ability to remember the contextual or detailed features of an event (i.e., source memory). In the perspective of the dual-process theories, item and source memory rely on different mnemonic processes, with item memory primarily assessing the ability of familiarity-based recognition, while source memory basing on recollection [8]. The neocortex, i.e., the prefrontal lobe and the parietal lobe, as well as the hippocampus and surrounding medial temporal lobes plays a prominent role in episodic memory. A great number of studies agreed that the interaction between medial temporal lobe and prefrontal cortex contributes to successful memory encoding and retrieval [8, 9]. Especially, the dorsolateral prefrontal cortex (DLPFC) is the crucial neural basis for episodic retrieval, involved in particularly complex retrieval process, such as information organization, postretrieval monitoring, and control-related modulation on the hippocampus [10, 11]. It is worth noting that although both item and source memory processes engage the neocortex, whether they rely on shared or distinct neural substrates is still up for debate.

Episodic memory is vulnerable to aging [12]. Impairments in episodic memory are at least partly attributed to structural and functional alterations in the brain accompanying the aging process [13, 14]. For instance, activation is frequently increased in the prefrontal cortex during the performance of memory tasks in older people, likely reflecting a compensatory mechanism for age-related cognitive deficits [15]. Whole-brain functional networks in healthy older adults also exhibit reorganization linked to decline memory performance [16, 17].

Fortunately, a growing body of literature has described the role of atDCS in improving episodic memory, including both item and associative memory, in older adults [18–21]. According to the latest meta-analysis, older adults receiving atDCS showed memory improvements, with a significant and modest effect size (Hedges′ *g* = 0.625, [22]). Beneficial effects of atDCS on episodic memory may result from modify learning-related synaptic connections and then modulating brain functions. However, there are still inconsistent results in the efficacy of tDCS on memory function [23, 24]. Moreover, the effects of atDCS on neural activity associated with episodic memory in older adults were rarely investigated, which would contribute to explaining the inconsistent results.

In the present study, multiple sessions of atDCS and functional magnetic resonance imaging (fMRI) were applied to assess the effects of stimulation on episodic memory and brain activity when engaging in memory retrieval. In this randomized, sham-controlled study, healthy older adults underwent fMRI scanning before and after atDCS treatment. Stimulation targeted the left dorsolateral prefrontal cortex (DLPFC), the crucial region for memory retrieval [25]. We aimed to investigate the neural mechanisms underlying atDCS-induced memory improvement. We hypothesized that (1) tDCS would enhance performance of episodic memory in older adults; (2) brain activity in the DLPFC during memory retrieval would evince alternation linked to memory improvement.

## 2. Materials and Methods

### 2.1. Study Design Overview

A randomized, single-blind, sham-controlled study was conducted. Healthy older adults received 10 daily sessions (30 min/day) of atDCS. Before and after the treatment with atDCS, participants were administered a demographic questionnaire and a battery of neuropsychological tests. The neuropsychological battery included the Montreal Cognitive Assessment—Beijing Version (MoCA-BJ) (Yu et al., 2012), the Center for Epidemiologic Depression Scale (CES-D) [26], the Paired Associative Learning Test (PALT) from the Clinical Memory Scale [27], the Auditory Verbal Learning Test (WHO-UCLA version) [28], the Digit Span Test [29], and Trail-Making Test (TMT) A and B [30], as well as the Category Fluency Test [31].

Before and after the treatment with atDCS, participants underwent fMRI scans during rest and performance of an episodic memory task. In the current study, the impacts of long-term stimulation on episodic-memory performance and corresponding neural functional changes were focused on.

This study is part of the project “Improving episodic memory with transcranial direct current stimulation and its neural correlates.” The project protocol was approved by the Ethics Committee of the Institute of Psychology, Chinese Academy of Sciences, and registered in the Chinese Clinical Trial Registry (ChiCTR) with the identifier ChiCTR-INR-16010036 (http://www.chictr.org/). All participants signed informed consent documents before taking part in the experiments.

### 2.2. Participants

Participants were randomly assigned to either an anodal or a sham tDCS group. The randomization sequence was generated by a third party who did not participate in this study using the online randomization generation tool. The allocation ratio was 1 : 1. The sample size planning was conducted. G∗Power 3.1[32] was used to determine sample size based on a medium effect size and power (1-*β* err prob) of 0.80. The results indicated that at least 17 participants per group were needed to detect a significant Group (the atDCS group vs.the sham group) × Test time (pretest vs. posttest) interaction at the 5% alpha level.

The inclusion criteria were as follows: (1) age ≥ 60 years, (2) education ≥ 9 years, (3) normal global cognitive function (a score of ≥21 on the MoCA-BJ), (4) normal emotional state (a score of ≤16 on the CES-D), and (5) no history of neurological or psychiatric disorders or traumatic brain injury. Sixty-four healthy older volunteers were initially recruited in and completed the project. Only those willing and eligible to participate in the fMRI sessions were included in the current study. Fifteen volunteers did not complete the fMRI sessions because they were unwilling to be scanned (*N* = 4), were ineligible for scanning (e.g., had metal implants; *N* = 8), could not understand the memory task (*N* = 3), or because of equipment failure (*N* = 1). Consequently, a total of 49 participants were included in the current study, with 24 in the anodal tDCS group and 25 in the sham tDCS group.

### 2.3. tDCS Protocol

A low, constant current was delivered using a battery-driven stimulator, the DC-STIMULATION MC8 (NeuroConn, Ilmenau, Germany), through a pair of 5 × 5 cm saline-soaked sponge electrodes. The target (anodal) electrode was placed on the scalp above the left DLPFC, corresponding to F3 in the 10-20 international EEG system [33] with an EEG cap. The reference (cathodal) electrode was placed on the right deltoid muscle to avoid an inhibitory effect on the brain.

A 2 mA constant direct current was delivered for 30 min, with 20 s ramp-on/ramp-off time at the beginning and end of the stimulation. The current density was 0.08 mA/cm^2^ and within safety limits [34, 35]. For sham stimulation, the electrodes remained in the same positions for 30 min, but the current lasted for 30 s each at the beginning and end of the stimulation to produce a physical sensation identical to the active stimulation. Each participant came to the laboratory for 10 consecutive days to receive the stimulation treatment. When receiving stimulation, participants were required to maintain sobriety and not do anything that demanded a high cognitive load.

After the final session of stimulation, the participants completed a brief poststudy questionnaire [36] regarding their subjective experience to evaluate the adverse effects. They rated the levels of adverse feelings (i.e., itchiness, skin pain, hotness, tingling, or other) on a 5-point Likert scale, with 1 signifying not feeling the sensation at all and 5 signifying feeling the sensation strongly. They also evaluated the duration and influence of these side effects on task performance.

### 2.4. Source Memory Task in the MRI Scanner

#### 2.4.1. Stimuli

A pool of 496 color pictures of objects divided into two sets was used as stimuli in the pretest and posttest scanning sessions. The assignment of these two sets to each scanning session was counterbalanced. An additional set containing 52 pictures was used for practice. The stimuli were arranged in pseudorandom order such that participants would not press the same response key on more than three consecutive trials. All stimuli appeared centrally within a white frame on a black background and subtended a visual angle of 5.4 × 5.4.

#### 2.4.2. Procedure

Participants practiced outside the scanner until they were familiar with the task. The experiment included four study blocks and four test blocks, conducted alternately. Short rests between blocks were self-paced. Both the study and test phases were scanned, but only the test data are reported here. Participants underwent an identical experiment with different sets of stimuli before and after the tDCS treatment. The posttest scanning session was usually scheduled in one week following the treatment, and the interval between the two scanning sessions was 15 days on average (range, 10–25 days).

### 2.5. Study

The stimuli in each study block consisted of 40 critical pictures plus two pictures as fillers at the beginning. During the study phase, each stimulus was displayed for 5,000 ms, with the cue words “Size” or “Living” below the picture. The participants decided whether the object would fit into a shoebox for the former cue or whether the object was living for the latter cue. Then, a fixation cross appeared for 1,000 ms, 3,000 ms, or 5,000 ms in a pseudorandom order, followed by a new trial. The participants responded with a button press with the two index fingers, providing a “bigger than a shoebox” or “living” response with one hand and a “would fit in a shoebox” or “nonliving” response with the other hand. Additionally, the participants were instructed to intentionally memorize pictures and corresponding cue words as pairs for the next test block.

#### 2.5.1. Test

**Figure 1:**
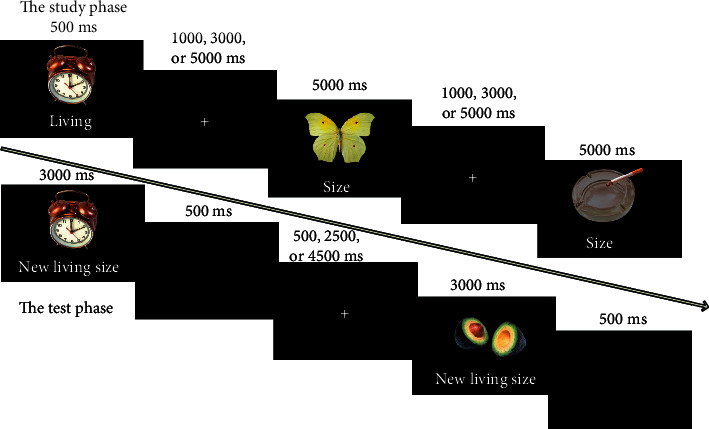
Schematic description of the study and test phases of the experiment.

Each test block was conducted immediately following a study block. The stimuli of each test block comprised 42 studied pictures (two were fillers) and 20 new pictures, with three cue words beneath the image corresponding to three responses: “New,” “Size,” and “Living.” Each stimulus was presented until the participants responded or until 3,000 ms had passed and was followed by a 500 ms black screen. A fixation cross was then presented for 500 ms, 2,500 ms, or 4,500 ms, after which the next trial began. If the participants judged that a picture had been encountered during the last study phase, they were required to provide a “Living” or “Size” response based on their response in the last study phase. If they thought a picture had not been shown in the previous study phase, or if they were not sure whether it was old or new, they were instructed to provide a “New” response. The participants responded by pressing keys on a magnet-compatible keyboard, with the middle and index fingers of one hand for “Size” and “Living” responses, respectively, and the index finger of the other hand for “New” responses. The assignment of hands to button presses was counterbalanced across the participants. See Figure 1 for the schematic of the experiment.

### 2.6. Behavioral Data Analyses

Statistical analysis was conducted with SPSS 21.0 (IBM Corp, Armonk, NY, USA). Group differences at baseline in cognitive and demographic variables were examined using the independent samples *t*-test and the chi-squared test for continuous and categorical data, respectively. Effects of stimulation on the source memory task and neuropsychological tests were examined by repeated measures ANOVA, with group (anodal tDCS vs. sham tDCS) as a between-subject factor and test time (pre vs. post) as a within-subject factor. Correlations between stimulation-related changes in brain activity and the memory performance were calculated using Pearson's correlation coefficient. Results with *p* < 0.05 were considered statistically significant.

Two critical outcome measures of the source memory task were calculated with the discrimination index Pr, which can exclude the contribution of random guesses (Snodgrass and Corwin, 1988). Specifically, the source and item Prs were computed by subtracting the false-alarm rates from the hit rates: source memory (Pr-source) was defined as the proportion of studied items whose source was correctly judged (source correct) minus the proportion of old items whose source was incorrectly judged, while item memory (Pr-item) was measured as the proportion of studied items judged as old—regardless of whether the source was correctly recognized—minus the false-alarm rate of new items.

### 2.7. FMRI Data Acquisition

A 3-Tesla GE scanner (GE Discovery MR750, GE Healthcare, Milwaukee, WI, USA) equipped for echo planar imaging at the MRI Research Center in the Institute of Psychology, CAS, was used for image acquisition. Initially, resting-state functional images were collected. During scanning, the participants were instructed to keep their eyes open, watch the fixation cross on the black screen, and remain relaxed with minimum movement. A total of 250 functional whole-brain volumes were acquired. Subsequently, the participants remained in the scanner and performed the memory task while task-related functional images were acquired. One hundred and seventy-one whole-brain volumes for each test session were acquired. The following parameters were used for functional images: resolution, 3.44 × 3.44 × 4 mm voxels; repetition time, 2,000 ms; echo time, 30 ms; flip angle, 90°; field of view, 220 × 220; slice gap, 0.5 mm; slice thickness, 3.5 mm; 37 axial slices; and acquisition matrix, 64 × 64 mm. High-resolution structural images were also acquired with a Sag 3D T1 BRAVO sequence with the following parameters: resolution, 1 × 1 × 1 mm voxels; inversion time, 450 ms; echo time, Min Full; flip angle, 8°; field of view, 256 × 256 mm; slice thickness, 1 mm; 176 sagittal slices; and acquisition matrix, 256 × 256 mm.

### 2.8. fMRI Data Analyses

Preprocessing and data analysis were performed using Statistical Parametric Mapping (SPM 12; Wellcome Department of Cognitive Neurology; http://www.fil.ion.ucl.ac.uk/spm/) and a toolbox for Data Processing & Analysis for Brain Imaging (DPABI, http://rfmri.org/) [37].

The first six image volumes of the first two trials of each block were discarded to allow for magnetization equilibrium and for the acclimatization of the subjects to the scanner. For each participant, functional images were subjected to preprocessing as follows: slice timing correction to the reference slice, spatial realignment for motion correction, coregistration with the participant's structural images, spatial normalization to the Montreal Neurological Institute (MNI) standard space followed by reslicing to a 3 × 3 × 3 mm resolution, and spatial smoothing using an 8 mm full-width-at-half-maximum Gaussian kernel. Two participants with head motion more than 3 mm translation or 3° rotation and one with poor quality of coregistration were excluded from further analyses.

In the first-level individual analysis, functional data were analyzed using the general linear model for each participant. In the design matrix for the statistical analysis, trial types as variables of interest consisted of retrieving the correct source information (source correct) and unsuccessfully retrieving the correct source information (source incorrect), successful recognition of old items collapsing the correct and incorrect source retrieval (item correct), and correct responses to new items (correct rejection). Movement parameters were also included as covariates of no interest. High-pass filtering was conducted using a cutoff period of 128 s to remove the low-frequency drifts from the time series. After model estimation, cerebral activation of source retrieval and item retrieval was estimated with the contrasts of “source correct vs. source incorrect trials” and “item correct vs. correct rejection trials,”, respectively.

Then, whole-brain group analyses were performed using the individual contrast images. First, with data of both groups obtained during the pretest, one-sample *t*-tests were performed to measure the areas of activation associated with source and item retrieval to determine whether the memory task activate classical retrieval-related brain networks, as found in previous studies. Second, we conducted repeated measures ANOVAs to identify brain regions that were significant in the Test time × Group interaction. A voxel-wise threshold of *p* < 0.005 and a *p* < 0.05 cluster-size threshold after correction for multiple comparisons, determined by Monte-Carlo simulation, were considered statistically significant.

## 3. Results

### 3.1. Demographic Characteristics and Results of Neuropsychological Tests

The demographics and results of neuropsychological tests for both groups are summarized in Table 1 (means ± standard deviation (SD)). The atDCS and sham groups did not differ significantly in age, sex, education, performance of episodic memory tests, or other cognitive tests before stimulation (all *p* > 0.05). After the 10 atDCS stimulations, 2 (group: anodal tDCS vs. sham tDCS) ∗2 (test time: pretest vs. posttest) repeated measures ANOVAs were conducted, and no significant interactions were revealed (all *p* > 0.05), suggesting that after stimulation, cognitive performance assessed with the neuropsychological tests did not change.

### 3.2. Brain Activation Related to Memory Retrieval (Before Stimulation)

The pretest activation maps for both source memory and item memory revealed widespread activity in the anterior parts of brain areas, including the middle frontal gyrus (MFG), anterior cingulate cortex (ACC), superior frontal gyrus (SFG), and the posterior areas of the precuneus and the inferior parietal lobule (IPL). In addition, bilateral hippocampus activation was pronounced in source memory but not in item memory (supplementary Figure S1 & Table S3). The activation patterns were similar to those shown to be involved in memory retrieval in previous studies [8, 38, 39].

### 3.3. Impact of Stimulation on the Source Memory Task

For the source memory task, the mean proportions of correct and incorrect source judgments and item judgments for old items and correct rejections to new items in the test phase, as well as reaction times corresponding to different response types for both groups are displayed in supplementary materials (Tables S1 and S2).

**Figure 2:**
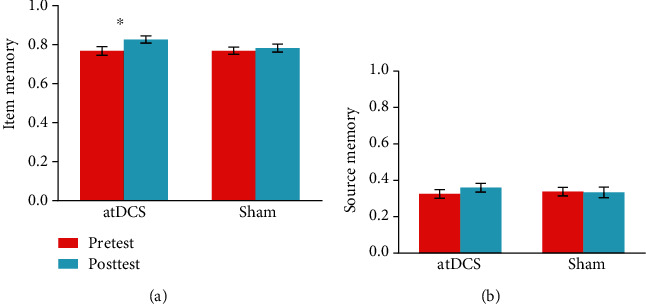
Item memory (a) and source memory (b) performance at pretest and posttest in the anodal transcranial direct current stimulation (atDCS) and sham groups. Error bars represent the standard error of the mean. ^∗^*p* < 0.05.

For Pr-items, the ANOVA revealed a Group × Test time interaction (*F*[1, 47] = 4.37, *p* = 0.037, *partial* *η*^2^ = 0.089). Further pairwise comparisons revealed significant performance improvement in the atDCS group (*p* < 0.001) but not in the sham group (*p* = 0.356), indicating that tDCS improved item memory (Figure 2). Meanwhile, the interaction in the index of Pr-source did not reach statistical significance (*F*[1, 47] = 1.02, *p* = 0.319, *partial* *η*^2^ = 0.021), although there was a trend for higher performance of source memory in the atDCS group than in the sham group.

Regarding adverse effects, all participants tolerated stimulation well, and none withdrew because of serious side effects. Each participant provided an average rating score on physical feelings, which showed no significant differences between the two groups (atDCS group: 1.64 ± 0.31; sham tDCS group: 1.50 ± 0.21; *t*[30] = 1.557; *p* = 0.130; Cohen′s *d* = 0.528).

### 3.4. Impact of Stimulation on Brain Activity during Memory Retrieval

**Figure 3:**
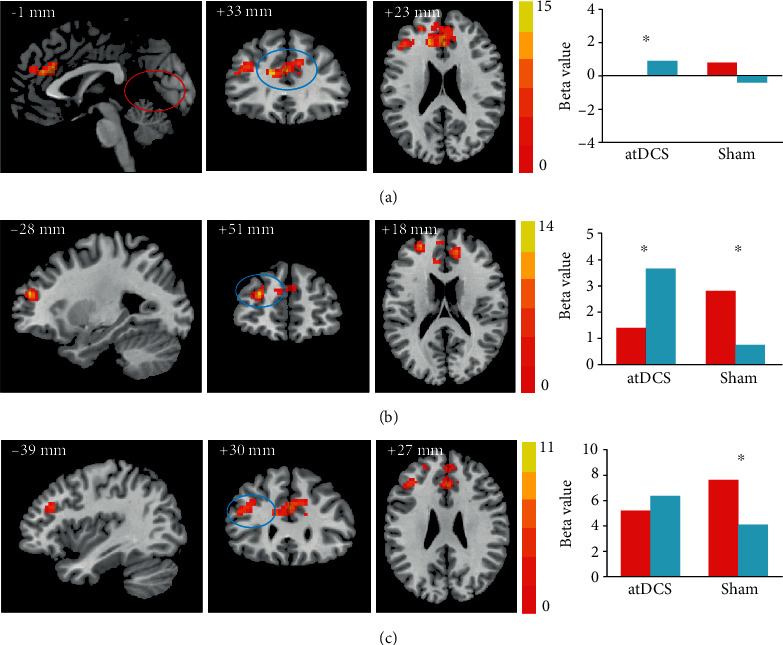
Visual maps for anodal transcranial direct current stimulation- (atDCS-) related activity alterations during item memory. Brain regions showed significant Group × Test time interactions in activation for (a) the bilateral anterior cingulate cortex (Brodmann area (BA) 32), (b) the left middle frontal gyrus 1 (BA 10), and (c) the left middle frontal gyrus 2 (BA 9/46, corrected for multiple comparisons with Monte-Carlo simulation, *p* < 0.05). Color bars are representative of *F* scores. Bar plots at the right show the mean beta values of brain activity in these regions before and after stimulation, for the atDCS and sham groups.

Whole-brain voxel-wise Group (anodal tDCS vs.sham tDCS) × Test time (pre vs. post) ANOVA was conducted to assess stimulation-induced activity differences in the contrast for source and item retrieval. First, in the contrasts of “source correct vs. source incorrect trials,” no cluster revealed a significant effect of atDCS on the neural functioning of source memory. Second, in the contrasts of “item correct versus correct rejection trials,” regions with significant interactions for Group × Test time were observed, mainly in the bilateral prefrontal areas. Specifically, there was one cluster located in the bilateral anterior cingulate cortex (ACC, Brodmann area (BA) 32; peak MNI location: -12 33 21; 215 voxels), and two clusters located in the left middle frontal gyrus (MFG1: BA 10; peak MNI location: -27 51 21; 37 voxels; MFG2: BA9/46; peak MNI location: -39 30 27; 40 voxels). Further, paired sample *t*-tests of the beta values extracted from these three regions were performed. Compared to the pretest, the atDCS group had selectively increased activity after stimulation in the bilateral ACC (*t*[23] = −2.90, *p* = 0.008, Cohen′s *d* = 0.675) and the MFG1 (*t*[23] = −2.30, *p* = 0.031, Cohen′s *d* = 0.382). The beta value in the MFG2 had a slightly increasing trend, which was not statistically significant (*p* > 0.05). Meanwhile, the sham group showed no significant changes in the ACC and significantly decreased activity in the MFG1 (*t*[21] = 2.75, *p* = 0.012, Cohen′s *d* = 0.419) and the MFG2 (*t*[21] = 3.03, *p* = 0.006, Cohen′s *d* = 1.707) in the posttest compared with the pretest. See Figure 3 for visualized activation maps and details regarding these regions.

**Figure 4:**
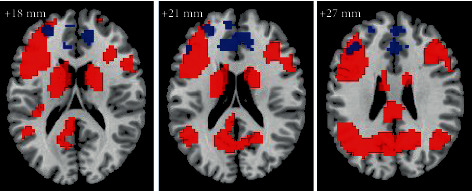
Brain regions activated in item memory before anodal transcranial direct current stimulation (atDCS) (red color) and showing significant Group × Test time interactions after atDCS (blue color). Overlap is present in the left middle frontal gyrus.

Furthermore, we explored whether atDCS enhanced activity in brain areas originally responsible for item memory or enhanced activity in new areas. Stacking together the activation map before stimulation (detailed in supplementary materials, Figure S1 and Table S3) and the map of activity alterations after stimulation in the item memory (Figure 4), we found that a small part of changed brain areas, including the MFG2 and part of the MFG1, overlapped with the activity areas involved in the task, while most brain regions, including the ACC and another part of the MFG1, showed increased activation located in new areas.

### 3.5. Relationship between Stimulation-Related Changes in Brain Activity and Behavioral Performance

We explored whether stimulation-related changes in brain activity during item memory retrieval were associated with item memory gains, by conducting Pearson product moment correlation analyses. Correlation analyses were carried out for the atDCS group and the sham group separately. We used [(posttest minus pretest)/pretest] to measure stimulation-related changes to exclude interference from the pretest.

**Figure 5:**
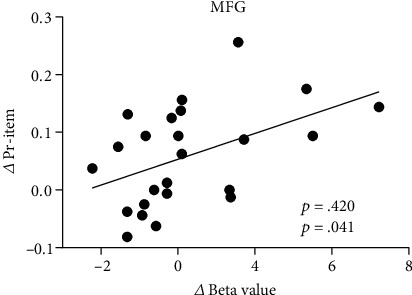
Correlation between the change in brain activity of the left middle frontal gyrus (MFG) and the change in item memory performance in the anodal transcranial direct current stimulation (atDCS) group. Each dot represents data from one participant. The regression line indicates a positive relationship.

Correlation analyses between stimulation-related changes in item memory performance and stimulation-related changes in brain activity during item memory retrieval in regions of interest (ROIs) were conducted. Three brain regions, including the ACC, MFG1, and MFG2, that showed significant Group × Test time interactions in brain activity of item memory were determined as ROIs. The beta values with a sphere of a 5 mm radius centered at the peak voxel with the maximal *F* value in ROIs were extracted. In the atDCS group, the results revealed a significantly positive correlation between the changes in MFG1 and item memory gains (*r* = 0.420, *p* = 0.041, Figure 5). However, the result was no longer significant with a Bonferroni correction threshold of 0.017 (0.05/3). In addition, there was also a trend of positive correlation in the ACC (*r* = 0.380, *p* = 0.067). Note that the sham group participants failed to show any correlation between the changes in item memory and brain activation (all *p* > 0.05).

## 4. Discussion

The present study examined whether multiple-session atDCS over the DLPFC could improve episodic memory and investigated the brain functional alterations that accompany such behavioral improvement. Behaviorally, stimulation enhanced item memory performance in an elderly population. FMRI results revealed that during item retrieval, there was significantly increased neural activity in the prefrontal areas and the ACC. Finally, correlation analyses revealed that item memory gains were positively correlated with increments in brain activity. Thus, it appears that atDCS can facilitate memory performance by modulating task-related activity in brain regions around and extending the stimulated sites.

### 4.1. Impact of Stimulation on Behavioral Performance

Regarding behavioral performance, atDCS over the DLPFC promoted item memory in the present study. Previously, several studies have shown atDCS-induced beneficial effects on item memory performance in healthy elderly participants (Brambilla et al., 2015; [19, 20, 40, 41]). In these studies, atDCS during memory encoding or retrieval (online stimulation) could facilitate memory performance. Our study extends the existing literature by revealing that offline atDCS with repeated sessions also has the potential to enhance episodic memory in healthy older adults.

Interestingly, source memory was not enhanced following atDCS administration in the intervention group. The finding suggests that the stimulation site of the left DLPFC might not be suboptimal to improve recollection-based retrieval. As previously described in Introduction, discrepant cognitive processes and separate neural substrates may underlie the processes of item and source recognition. Some research found that the DLPFC is responsible for familiarity, while the temporal lope is associated with both recollection and familiarity [42–44]. The activity of hippocampus related to these two processes was largely nonoverlapping, also revealing that two complementary but distinct mechanisms supporting episodic memory [45]. More importantly, activations in the parietal cortex mediated attention processes in complex memory retrieval, mainly affecting the events' contextual details [46, 47]. The parietal cortex showed greater activity when orienting attention toward source recollections compared to item recognition [48]. Consistently, in many previous studies [18, 49–53], tDCS targeting the temporoparietal area or the lateral parietal cortex led to superior recollection performance. In summary, existing research may indicate that stimulation over the temporoparietal area rather than the left DLPFC possibly is more effective for associative memory. Future research is needed to examine this possibility. We would acknowledge that as a new approach to enhance cognitive function; tDCS is still very preliminary. More comprehensive outcome measures are required to determine when and where tDCS can be applied to maximize memory performance in future studies.

### 4.2. Impact of Stimulation on Brain Activity during Memory Retrieval

Consistent with the behavioral results, we only found tDCS effects on brain functional activity during item memory retrieval. Increased activation in the left DLPFC and other MFG as well as the ACC was observed only in the atDCS group during posttest fMRI acquisition. More pronounced activity in these areas was associated with increased item memory performance. These findings revealed the possible underpinnings of tDCS effects on memory.

As expected, atDCS increased the blood-oxygen-level-dependent signal during item retrieval, in agreement with the physiology of tDCS. Subthreshold membrane depolarization is considered the physiological mechanism of atDCS. Following atDCS, spontaneous neuronal firing activity increases and then the excitability of neuronal populations increases [2, 54]. Additionally, the hemodynamic response of the stimulated cortical area changed, appearing as an increase in regional cerebral blood flow [55].

Moreover, the change in the activation pattern after atDCS is congruent with the widely recognized compensation hypothesis of aging. To date, a considerable amount of literature has described greater frontal recruitment across multiple cognitive tasks in older adults [15, 56]. Increased frontal recruitment is linked to improved cognitive performance in older adults, indicating that additional age-related prefrontal activation may be functional and beneficial to task performance. The scaffolding theory of aging and cognition [57] states that complementary and alternative neural circuits develop in the aging brain to counteract the age-associated progressive inefficiency of existing neural circuits. Consistent with this hypothesis, we found that neural activity in the prefrontal cortex and the ACC increased after atDCS, and the neural alterations were accompanied by improvement in behavioral performance. It is increasingly recognized that the ACC is engaged in a variety of cognitive and emotional processes, such as error detection and evaluation, conflict monitoring, response selection, and attention control [58]. Several lines of evidence indicate that the ACC also plays a pivotal role in continuous conflict monitoring during memory retrieval [59–61]. As a result, the ACC appears to serve as available scaffolding, playing a compensatory role for the decline in functional processing resources after atDCS.

Similarly, Holland et al. [62] and Meinzer et al. [63] also investigated how tDCS induced brain activity changes related to cognitive tasks (i.e., priming and language) in elderly populations. Interestingly, contrary to our results, task-related activity was reduced in the atDCS group compared to the sham group in both studies. Meinzer et al. [63] proposed that atDCS reversed age-associated hyperactivity in the prefrontal cortex and enhanced neural efficiency. However, behavioral priming and language ability, assessed in these studies, were less impaired in the ageing processing, comparing to memory. As a result, compensatory recruitment of novel brain regions may not have been necessary for these cognitive tasks in the aging brain. In parallel with our results, in studies using other methods of brain stimulation or cognitive training to improve memory performance, additional recruitment of novel brain regions has always been observed [64–66]. These findings imply that compensation is more readily available for severely damaged cognitive functions, such as episodic memory.

### 4.3. Limitations and Future Directions

This study is subject to several limitations. Although a postsimulation questionnaire was administered, in which adverse events were surveyed, whether blinding succeed was not asked. Because it could have exposed the experiment purpose and design and then interfered with the follow-up performance. The second major limitation is the absence of a younger control group. As a result, we could not provide direct evidence that atDCS mitigated the age-related cognitive decline through prefrontal compensatory mechanisms. Our discussion regarding compensation may therefore be regarded as speculative. In the future, a direct comparison between brain activity alterations in an atDCS group and a younger control group is warranted. Third, episodic memory in everyday life was not assessed. Memory plays important roles in everyday function. However, given the low ecological validity of the laboratory tasks, it is difficult to draw a conclusion that tDCS was beneficial to older adults in real life. In the future, well-designed memory outcomes and randomized controlled trials are expected to verify the impact of tDCS on everyday memory. Another issue not addressed in this study was the duration of the stimulation-induced changes in brain plasticity. Longitudinal studies will be required to evaluate tDCS effects on the neural plasticity of the aging brain over time.

## 5. Conclusion

To the best of our knowledge, this was the first study to investigate atDCS-induced changes in brain activity during memory retrieval and their relationship with episodic memory gains in an elderly population. The major finding that the effect of atDCS on item memory rather than source memory indicates that different stimulation protocols are beneficial to different memory processes, which have important implications for future clinical application of atDCS. Additionally, the results concerning brain plasticity suggest that strengthening regional neural activity around the stimulation areas may lead to enhanced memory function, contributing to the elucidation of the neural mechanisms underlying atDCS in healthy older adults. atDCS holds the potential to mitigate age-related memory decline, which is a major issue for aging societies worldwide.

## Figures and Tables

**Table 1:** Demographic characteristics and neuropsychological results at baseline for the stimulation and control groups.

	atDCS group (*N* = 24)	Sham group (*N* = 25)	*P*
Age	66.58 ± 6.11	65.48 ± 3.39	.436
Gender (F/M)	11/13	9/16	.201
Education (year)	12.50 ± 2.28	12.04 ± 2.03	.460
MoCA	26.42 ± 2.38	25.88 ± 1.74	.370
CES-D	6.92 ± 4.63	3.80 ± 2.96	.007
DSF	7.21 ± 1.47	7.00 ± 1.19	.588
DSB	5.13 ± 1.39	4.44 ± 1.33	.085
TMT-A	31.76 ± 8.76	30.97 ± 10.06	.772
TMT-B	77.05 ± 41.17	60.27 ± 19.60	.080
Category fluency test	44.38 ± 9.67	45.12 ± 8.47	.775
PALT	8.98 ± 2.49	9.72 ± 3.03	.355
AVLT-IR	6.54 ± 2.34	6.16 ± 1.93	.536
AVLT-DR	11.25 ± 2.45	12.28 ± 1.86	.104
AVLT-learning	49.58 ± 10.69	52.72 ± 7.40	.237

atDCS: anodal transcranial direct current stimulation; CES-D: Center for Epidemiologic Depression Scale; MoCA: Montreal Cognitive Assessment; DSF: digit span forward; DSB: digit span backward; TMT: trail-making test; PALT: paired associative learning test; AVLT: auditory verbal learning test; IR: immediate recall; DR: delayed recall; *P*: significant level of independent *t*-test between the two groups at baseline.

## Data Availability

The behavioral and fMRI data used to support the findings of this study are available from the corresponding author upon request.

## Supplementary Materials

Supplementary Materials Supplementary Table S1: mean accuracy for each response type in the anodal transcranial direct current stimulation (atDCS) and sham groups (mean ± SD). Supplementary Table S2: mean reaction time for each response type in the anodal transcranial direct current stimulation (atDCS) and sham groups (mean ± SD). Supplementary Table S3: memory-related activity at pretest. Supplementary Figure S1: activation maps during pretests of all participants in the following contrasts: source correct vs. source incorrect (A), and item correct vs. correct rejection (B), before the intervention of transcranial direct current stimulation. All analyses were corrected for multiple comparisons using Monte Carlo simulation (voxel level *p* < 0.001, cluster *p* < 0.05). Color bars below maps show *t*-values.
