# Supplementary material for: Transcranial Direct Current Stimulation Enhances Episodic Memory in Healthy Older Adults by Modulating Retrieval-Specific Activation
Source: Neural Plast. 2020 Dec 5;2020:8883046. doi: 10.1155/2020/8883046 (PMC7735856; doi:10.1155/2020/8883046)
Supplement: Supplementary Materials — Supplementary Table S1: mean accuracy for each response type in the anodal transcranial direct current stimulation (atDCS) and sham groups (mean ± SD). Supplementary Table S2: mean reaction time for each response type in the anodal transcranial direct current stimulation (atDCS) and sham groups (mean ± SD). Supplementary Table S3: memory-related activity at pretest. Supplementary Figure S1: activation maps during pretests of all participants in the following contrasts: source correct vs. source incorrect (A), and item correct vs. correct rejection (B), before the intervention of transcranial direct current stimulation. All analyses were corrected for multiple comparisons using Monte Carlo simulation (voxel level p < 0.001, cluster p < 0.05). Color bars below maps show t-values. [file 8883046.f1.docx]

Supplementary materials

**Supplementary Table S1**. Mean accuracy for each response type in the anodal transcranial direct current stimulation (atDCS) and sham groups (mean ± SD).

|  | atDCS Group (N = 24) | | Sham Group (N = 25) | | *p* | |
| --- | --- | --- | --- | --- | --- | --- |
|  | Pre | Post | Pre | Post |  |  |
| **Old items** |  |  |  |  |  | |
| Source correct | 0.58 ± 0.09 | 0.62 ± 0.08 | 0.59 ± 0.09 | 0.59 ± 0.10 | .113 | |
| Source incorrect | 0.25 ± 0.05 | 0.26 ± 0.06 | 0.25 ± 0.06 | 0.26 ± 0.06 | .967 | |
| Item correct | 0.83 ± 0.08 | 0.87 ± 0.08 | 0.84 ± 0.09 | 0.85 ± 0.09 | .023 | |
| Item miss | 0.17 ± 0.08 | 0.12 ± 0.08 | 0.15 ± 0.09 | 0.14 ± 0.08 | .028 | |
| **New items** |  |  |  |  |  | |
| Correct rejection | 0.88 ± 0.10 | 0.90 ± 0.10 | 0.86 ± 0.13 | 0.87 ± 0.13 | .693 | |
| **memory indices** |  |  |  |  |  | |
| Source memory (Pr-Source) | 0.33 ± 0.12 | 0.36 ± 0.12 | 0.34 ± 0.12 | 0.33 ± 0.15 | .319 | |
| Item memory (Pr-Item) | 0.77 ± 0.11 | 0.83 ± 0.09 | 0.77 ± 0.09 | 0.78 ± 0.10 | .037 | |

**Supplementary Table S2.** Mean reaction time for each response type in the anodal transcranial direct current stimulation (atDCS) and sham groups (mean ± SD).

|  | atDCS Group (N = 24) | | Sham Group (N = 25) | | *p* |
| --- | --- | --- | --- | --- | --- |
|  | Pre | Post | Pre | Post |  |
| **Old items** |  |  |  |  |  |
| Source correct | 1778 ± 197 | 1767 ± 211 | 1793 ± 271 | 1707 ± 286 | .096 |
| Source incorrect | 1915 ± 222 | 1916 ± 192 | 1939 ± 283 | 1895 ± 348 | .418 |
| Item correct | 1847 ± 203 | 1842 ± 194 | 1866 ± 271 | 1801 ± 309 | .183 |
| Item miss | 1663 ± 245 | 1582 ± 355 | 1706 ± 288 | 1725 ± 351 | .240 |
| **New items** |  |  |  |  |  |
| Correct rejection | 1366 ± 199 | 1409 ± 282 | 1443 ± 264 | 1392 ± 243 | .013 |

**Supplementary Table S3.** Memory-related activity at pretest

| Brain areas | R/L | BA | Maxima of cluster | | | | | *t* value | Cluster size (voxels) |
| --- | --- | --- | --- | --- | --- | --- | --- | --- | --- |
|  |  |  | x | y | | z | |  |  |
| **Source correct > source incorrect** |  |  |  | |  | |  |  |  |
| OL/Precuneus/IPL | R,L | 5,7,18,39 | 9 | | -69 | | -15 | 8.60 | 5802 |
| MFG/SFG/ACC | L | 9,10,32 | -6 | | 45 | | 6 | 7.05 | 1425 |
| Hippocampus/STG | R | 20,24 | 24 | | -18 | | -9 | 6.38 | 266 |
| Hippocampus | L | 20 | -30 | | -18 | | -9 | 4.44 | 105 |
| **Source incorrect > source correct** |  |  |  | |  | |  |  |  |
| None |  |  |  | |  | |  |  |  |
| **Item correct > correct rejection** |  |  |  | |  | |  |  |  |
| OL/Precuneus/IPL/MFG | R,L | 6,7, 39,40,46 | -33 | | -66 | | 42 | 19.78 | 11807 |
| MFG/SFG | R | 10 | 27 | | 57 | | 6 | 6.50 | 152 |
| **Correct rejection > Item correct** |  |  |  | |  | |  |  |  |
| MFG/ACC | R,L | 32 | 3 | | 33 | | 6 | -5.17 | 183 |
| STG/Insula | L | 13,48 | -39 | | -21 | | 3 | -5.95 | 206 |

Notes: BA: Brodmann area; OL: occipital lobe; IPL, inferior parietal lobule; MFG: middle frontal gyrus; SFG: superior frontal gyrus; ACC: anterior cingulate cortex; STG: superior temporal gyrus


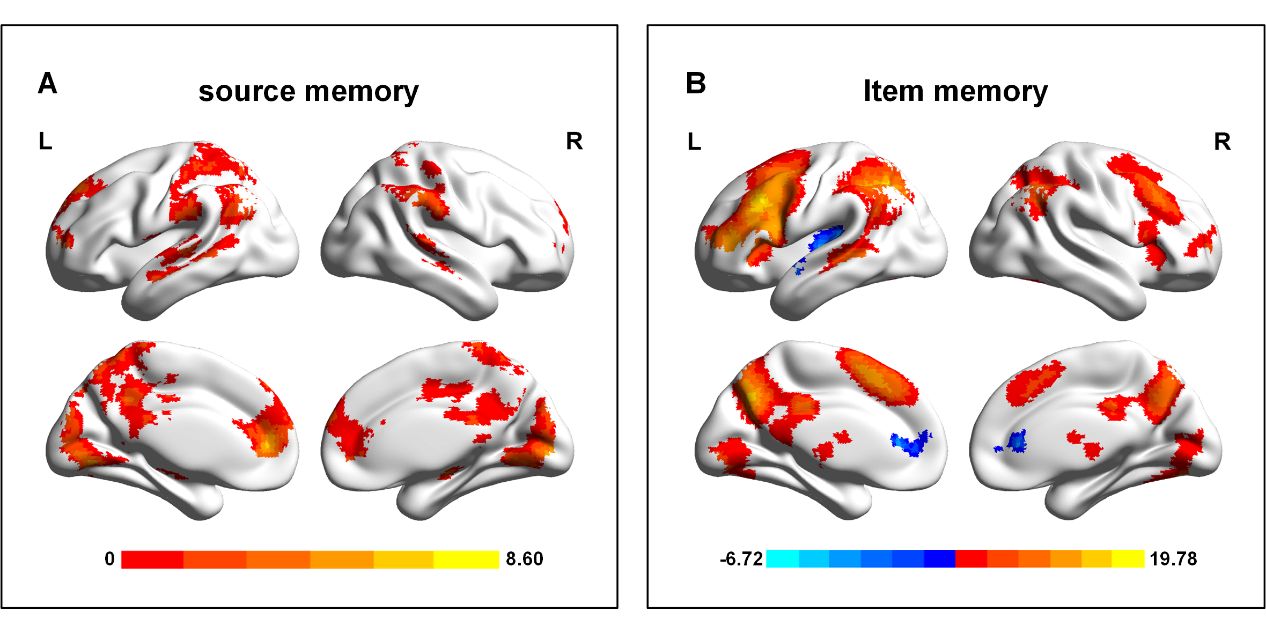


**Supplementary Figure S1.** Activation maps during pretests of all participants in the following contrasts: source correct vs. source incorrect (A), and item correct vs. correct rejection (B), before the intervention of transcranial direct current stimulation. All analyses were corrected for multiple comparisons using Monte Carlo simulation (voxel level *p* < 0.001, cluster *p* < 0.05). Color bars below maps show t-values.
